# Effect of Anti-Diabetic Medication Use on Sepsis Risk in Type 2 Diabetes Mellitus: A Multivariate Analysis

**DOI:** 10.3390/geriatrics10040108

**Published:** 2025-08-07

**Authors:** Battamir Ulambayar, Amr Sayed Ghanem, Attila Csaba Nagy

**Affiliations:** 1 Department of Health Informatics, Faculty of Health Sciences, University of Debrecen, 4032 Debrecen, Hungary; ulambayar.battamir@etk.unideb.hu (B.U.); aghanem@etk.unideb.hu (A.S.G.); 2 Coordinating Centre for Epidemiology, University of Debrecen Clinical Centre, 4032 Debrecen, Hungary

**Keywords:** type 2 diabetes mellitus, sepsis, insulin, sodium–glucose cotransporter-2 (SGLT2) inhibitors, glucagon-like peptide-1 (GLP-1) receptor agonists

## Abstract

**Background:** Type 2 diabetes mellitus (T2DM) increases sepsis risk due to immune dysfunction and chronic inflammation. Antidiabetic medications, while primarily used for glycemic control, may modulate sepsis susceptibility through immune and inflammatory pathways. This study investigates the association between antidiabetic medication use and sepsis risk in T2DM patients. **Methods:** A longitudinal cohort study was conducted using clinical registry data from 5009 T2DM patients at the University Hospital, Debrecen, Hungary (2016–2020). Sepsis cases were identified via ICD-10 code A41, and antidiabetic medication use was categorized using ATC codes. Baseline comorbidities and laboratory parameters were extracted. Chi-square and Wilcoxon rank–sum tests assessed associations between sepsis and categorical/numerical variables, respectively. Time-adjusted multivariate logistic regression evaluated predictors of sepsis risk, with odds ratios (ORs) and 95% confidence intervals (CIs) reported. **Results:** Age, hypertension, ischemic heart disease, nephropathy, elevated blood glucose, C-reactive protein, and creatinine also independently increased sepsis risk. Insulin use was associated with a 2.6-fold increased sepsis risk (OR = 2.6, 95% CI: 2.09–3.34, *p* < 0.001), while SGLT2 inhibitors (OR = 0.56, 95% CI: 0.34–0.91, *p* = 0.02) and GLP-1 receptor agonists (OR = 0.39, 95% CI: 0.19–0.79, *p* = 0.009) were protective. **Conclusions:** Insulin-treated patients may require closer infection monitoring, while SGLT2 inhibitors and GLP-1 RAs could be prioritized in high-risk individuals. These findings highlight the potential to inform risk stratification and guide personalized antidiabetic therapy to reduce sepsis risk in T2DM.

## 1. Introduction

Type 2 diabetes mellitus (T2DM) is a prevalent chronic metabolic disease that affects over 400 million individuals globally [1] and is characterized by insulin resistance, impaired insulin secretion, and hyperglycemia [2]. Beyond its well-documented effects on macro and microvascular complications, T2DM significantly increases susceptibility to infections, including sepsis, a life-threatening syndrome caused by a dysregulated immune response to infection [3]. Sepsis remains a leading cause of hospitalization and mortality worldwide, with an estimated 50 million cases annually and a mortality rate ranging from 20% to 50% [4], particularly in high-risk populations such as those with T2DM [5]. The increased infection risk in T2DM is attributed to immune dysfunction, including impaired neutrophil activity, altered cytokine production, and chronic low-grade inflammation, all of which may exacerbate the systemic inflammatory response in sepsis [5].

Antidiabetic medications are the cornerstone of T2DM management, aimed at achieving glycemic control and mitigating long-term complications. These include traditional agents such as insulin, biguanides, and sulfonylureas and newer classes like sodium–glucose cotransporter-2 (SGLT2) inhibitors, glucagon-like peptide-1 (GLP-1) receptor agonists, and dipeptidyl peptidase-4 (DPP-4) inhibitors [6]. While these medications are primarily evaluated for their glucose-lowering efficacy [7,8], emerging evidence suggests that they may influence immune responses and inflammatory processes through multiple mechanisms [9]. For instance, metformin has been shown to possess anti-inflammatory properties, potentially reducing pro-inflammatory cytokine levels [10], whereas SGLT2 inhibitors may influence immune responses through mechanisms independent of glycemic control, such as alterations in gut microbiota or ketone metabolism [6]. Insulin has anti-inflammatory effects and can modulate immune cell function, suggesting that it plays an important immunomodulatory role [11].

Despite the critical intersection of T2DM, antidiabetic therapy, and infection risk, the specific impact of these medications on sepsis susceptibility remains poorly understood. First, studies suggest that poor glycemic control correlates with increased sepsis incidence and mortality [12,13]. Conversely, appropriate management of T2DM can improve immunological adaptability, thereby lowering the risk of sepsis-related morbidity and mortality [14]. This highlights the vital need to include glycemic control measures as a variable in studies evaluating the effects of antidiabetic drugs on sepsis risk. As mentioned above, antidiabetic medications have been shown to have protective effects against infections; however, the results are not always consistent, as some studies report no significant differences [15] or even suggest an increased risk of sepsis with certain medications [16]. Therefore, it is important to explore the specific impacts of various antidiabetic medications on sepsis risk.

This study aims to address these knowledge gaps by conducting a multivariate analysis of the association between the most commonly used antidiabetic medication and sepsis risk in a large, longitudinal cohort of patients with T2DM. By examining the effects of individual medication classes while controlling for key clinical variables, this research seeks to reveal their independent contributions to sepsis susceptibility in this vulnerable population.

## 2. Materials and Methods

### 2.1. Study Design and Data

In this longitudinal study, we used the clinical registry database of patients at the Department of Internal Medicine of the University Hospital, Debrecen, in Hungary between 2016 and 2020. We included adult patients (aged ≥18 years) with confirmed diagnoses of T2DM, identified based on ICD codes (E11) and prescription records, who each had at least one documented outpatient prescription for antidiabetic medications between 2016 and 2020. Patients were required to have available follow-up data for the observation period. Patients with missing key demographic data (e.g., age, sex), incomplete clinical records, or a history of sepsis before the start of follow-up were excluded. The data were fully anonymized before analysis. As this was a retrospective study, traditional blinding was not applicable. However, to reduce bias, data analysts worked only with de-identified data and were not involved in data collection.

### 2.2. Variables

In total, 5009 patients diagnosed with T2DM according to clinical guidelines were included in the dataset. The cases of sepsis, the primary outcome of the cohort, were determined by filtering the ICD-10 code A41 for each year. Binary variables were also created using Anatomical Therapeutic Chemical (ATC) codes to filter for antidiabetic drugs used in that year to indicate whether the treatment was administered. From the patients’ annual diagnostic records, binary variables were created using the appropriate ICD-10 codes to identify whether diabetes comorbidities such as hypertension, ischemic heart disease, dyslipidemia, atherosclerosis, nephropathy, and neuropathy were diagnosed in that year. The data contain the key clinical laboratory parameters measured yearly as well. The dataset was sorted by patient ID and year as longitudinal data for the statistical analysis.

### 2.3. Statistical Analysis

The chi-square test was used to determine the association between the incidence of sepsis and baseline category variables among patients with T2DM. Because numerical variables such as age, BMI, and clinical laboratory parameters were not normally distributed, the Wilcoxon rank–sum test was used to test the hypothesis that these variables were different between patients with and without sepsis. Based on the statistically significant associations identified, time-adjusted multivariate logistic regression was used to identify factors affecting the risk of sepsis in patients with T2DM. As the outcome of interest—sepsis—was evaluated as a binary event occurring at annual intervals with minimal censoring and no precise time-to-event data, the assumptions required for Cox regression (e.g., proportional hazards and event timing) were not met. Logistic regression was more appropriate given the structure of our longitudinal dataset and allowed us to assess associations while adjusting for annual follow-up time as a covariate. We also confirmed the model’s strong discriminative power using AUC, which further supported our methodological approach. The odds ratio (OR) and 95% confidence interval (CI) were used to express the outcomes of logistic regression analysis, and a *p*-value lower than 0.05 was considered to be statistically significant. STATA IC version 18.0 was used to conduct the statistical analyses in this study [17].

### 2.4. Ethical Approval and Informed Consent

The Ethics Committee of the University of Debrecen approved the studies involving secondary data analysis of human participant data, ensuring compliance with ethical standards and the Declaration of Helsinki. These studies were conducted in accordance with local legislation, institutional requirements, and national guidelines for research involving human data. Approval was granted by the Ethics Committee of the University of Debrecen under protocol number 5610–2020. As the research involved secondary analysis of anonymized data, obtaining individual informed consent from participants was not required. The data were fully de-identified and handled in compliance with applicable data protection regulations to ensure the confidentiality and privacy of study subjects.

## 3. Results

### 3.1. Baseline Characters of the Patients

The baseline characteristics of patients with T2DM enrolled in this study are shown in Table 1. The median age was 66 years, with an approximately equal distribution of female (50.9%, *n* = 2550) and male (49.1%, *n* = 2459) patients. The median BMI was 30.4 kg/m^2^ (IQR: 26.1–35.7), indicating that the majority of patients were overweight or obese. Comorbidities were prevalent in the cohort, with hypertension being the most common (62.7%, *n* = 3141), followed by ischemic heart disease (39.1%, *n* = 1956), dyslipidemia (29.2%, *n* = 1463), and atherosclerosis (27.7%, *n* = 1388). Nephropathy and neuropathy were observed in 22.3% (*n* = 1113) and 12.4% (*n* = 622) of patients, respectively, reflecting a significant burden of diabetes-related complications at baseline. Antidiabetic medication use varied, with metformin being the most frequently prescribed (34.9%, *n* = 1747), followed by insulin (23.5%, *n* = 1179). SGLT-2 inhibitors and GLP-1 RAs were less commonly used. Biochemical markers indicated suboptimal glycemic control and systemic inflammation in the cohort. The median blood glucose was 7.8 mmol/L (IQR: 6.4–10.2), and the median HbA1c was 7.2% (IQR: 6.5–8.3). The median C-reactive protein level was 4.4 mg/L (IQR: 2.0–11.1), indicative of low-grade systemic inflammation. Lipid profiles showed a median total cholesterol of 4.6 mmol/L (IQR: 3.8–5.5) and median triglycerides of 1.6 mmol/L (IQR: 1.2–2.4). The median creatinine level was 80 µmol/L (IQR: 65–101), which is consistent with generally preserved renal function.

### 3.2. Comparison of Patients With and Without Sepsis

Table 2 presents a comparison of baseline characteristics between patients with T2DM who developed sepsis (*n* = 197) and those who did not (*n* = 4812). Patients with sepsis were significantly older, with a median age of 72 years (IQR: 64–79) versus 66 years (IQR: 60–73) in the non-sepsis group (*p* < 0.001). BMI was also significantly higher in the sepsis group (median: 34.5 kg/m^2^, IQR: 32.1–41.5) compared to those without sepsis (30.4 kg/m^2^, IQR: 26.1–35.6; *p* = 0.006). Regarding comorbidities, hypertension (5.7% vs. 1.2%, *p* < 0.001), ischemic heart disease (4.9% vs. 3.4%, *p* = 0.007), atherosclerosis (5.3% vs. 3.4%, *p* = 0.002), and especially nephropathy (10.1% vs. 2.2%, *p* < 0.001) were significantly more prevalent in the sepsis group, indicating a higher burden of cardiovascular and renal complications. In terms of antidiabetic medications, insulin use was markedly higher among patients with sepsis (7.3% vs. 2.9%, *p* < 0.001), while none of the sepsis patients received GLP-1 receptor agonists, compared with 4.1% in the non-sepsis group (*p* = 0.004) who received them.

Laboratory parameters also distinguished the groups. Median blood glucose was slightly higher in the sepsis patients (8.2 mmol/L, IQR: 6.4–10.7) versus 7.8 mmol/L (IQR: 6.3–10.1) in the non-sepsis patients (*p* = 0.006). CRP levels were drastically elevated in the sepsis group (60.1 mg/L, IQR: 32.3–103.9) compared with the non-sepsis group (4.1 mg/L, IQR: 1.9–9.8; *p* < 0.001), suggesting substantial systemic inflammation. Additionally, the total cholesterol (4.1 vs. 4.6 mmol/L, *p* < 0.001) and triglyceride levels (1.4 vs. 1.6 mmol/L, *p* < 0.001) were lower in the sepsis patients. Creatinine levels were significantly higher in the sepsis group (99 µmol/L, IQR: 67–161) compared with those without sepsis (80 µmol/L, IQR: 65.5–100; *p* < 0.001), indicating possible renal impairment.

Overall, these results highlight that patients with T2DM who developed sepsis were older, more obese, had more frequent cardiovascular and renal comorbidities, had higher insulin use, and showed stronger inflammatory and renal dysfunction markers than those without sepsis.

### 3.3. Multivariate Analysis

The results of a time-adjusted multivariate logistic regression model evaluating predictors of sepsis in patients with T2DM are shown in Table 3. Each one-year increase in age was associated with 2% increased odds of sepsis (OR = 1.02, 95% CI: 1.01–1.03, *p* = 0.002). Hypertension significantly increased the odds of sepsis by 3.4-fold (OR = 3.4, 95% CI: 2.1–5.5, *p* < 0.001), while ischemic heart disease and nephropathy were associated with 56% (OR = 1.56, 95% CI: 1.22–2.0, *p* = 0.001) and 98% (OR = 1.98, 95% CI: 1.50–2.61, *p* < 0.001) increased odds, respectively. Insulin use was associated with a 2.6-fold increased odds of sepsis (OR = 2.6, 95% CI: 2.09–3.34, *p* < 0.001), whereas SGLT-2 inhibitors and GLP-1 RAs were protective, reducing the odds of sepsis by 44% (OR = 0.56, 95% CI: 0.34–0.91, *p* = 0.02) and 61% (OR = 0.39, 95% CI: 0.19–0.79, *p* = 0.009), respectively. Biochemical markers also showed significant associations: each 1 mmol/L increase in median blood glucose increased the odds of sepsis by 7% (OR = 1.07, 95% CI: 1.03–1.1, *p* < 0.001), each 1 mg/L increase in median CRP increased the odds by 4% (OR = 1.04, 95% CI: 1.03–1.05, *p* < 0.001), and each 1 µmol/L increase in median creatinine increased the odds by 2% (OR = 1.02, 95% CI: 1.01–1.03, *p* < 0.001). The time-adjusted multivariate logistic regression model demonstrated strong discriminative performance in predicting sepsis among patients with T2DM, with an AUC of 0.819 (95% CI: 0.802–0.837).

## 4. Discussion

This longitudinal study found that insulin use was associated with a 2.6-fold increase in sepsis risk among patients with T2DM, even after adjusting for age, comorbidities, and lab markers. Conversely, SGLT-2 inhibitors and GLP-1 receptor agonists were independently associated with lower sepsis risk. Older age [18,19], cardiovascular comorbidities [20,21,22,23,24,25], and nephropathy [26,27,28,29,30] emerged as significant predictors of sepsis risk, suggesting these subgroups within T2DM warrant intensified infection prevention strategies.

Our findings suggest that patients who use insulin are at increased risk of sepsis, which may be primarily explained by the fact that insulin therapy typically indicates more severe or advanced cases of T2DM [31], placing these patients at inherently higher risk of infections and sepsis. Insulin therapy has been implicated in an increased risk of severe hypoglycemia, which can adversely affect immune responses and contribute to poorer outcomes during infections [32]. Several studies highlight the fact that insulin may be associated with higher rates of infections, including sepsis, compared with other glucose-lowering agents [33]. This suggests a potential mechanism where hyperglycemia-related inflammation and poor glycemic control create an environment conducive to infections [34].

However, the relationship between insulin therapy and sepsis risk is complex and influenced by multiple factors. Patients with T2DM generally have impaired immune responses, increasing their susceptibility to infections and sepsis. Insulin therapy improves glycemic control, which is essential for optimal immune function, and insulin itself has anti-inflammatory effects by the suppression of key inflammatory molecules and the modulation of immune cell activity [15], which are manifested by its actions in suppressing key inflammatory molecules and modulating the activity of immune cells [35]. However, there is concern that insulin therapy may increase the risk of severe infections due to its anti-inflammatory effects and metabolic abnormalities. In particular, hyperinsulinemia is associated with a prothrombotic state characterized by increased levels of fibrinogen and plasminogen activator-1, which contributes to the development of infections and complications associated with sepsis [36]. Studies have shown that intensification of insulin therapy leads to episodes of hypoglycemia, which is associated with an increased risk of cardiovascular complications in this population, which increases the risk of sepsis [37]. Therefore, glycemic fluctuations during insulin therapy may trigger immune dysregulation, while its effects on inflammation and wound healing may inadvertently increase infection risk [38,39], and this evidence confirm our results.

Recent studies suggest that GLP-1 RAs may reduce the risk of sepsis in patients with T2DM. A large retrospective cohort study found that GLP-1 RA treatment was associated with a significant reduction in the risk of severe sepsis as compared to dipeptidyl peptidase-4 inhibitor (DPP-4i) [40]. The reason why GLP-1 RAs reduce the risk of sepsis in patients with T2DM may be primarily explained by the anti-inflammatory mechanism of this group of drugs. GLP-1 RA treatment has been shown to reduce pro-inflammatory cytokines such as tumor necrosis factor-alpha, interleukin-1β, and IL-6, and thereby increase anti-inflammatory adiponectin in patients with T2DM [41]. Other studies have also shown that GLP-1 RAs can suppress inflammation in patients with sepsis by improving organ systems’ functions by regulating blood sugar and inflammation [42]. Similarly, GLP-1 RAs have been shown to reduce inflammatory gene expression in patients with T2DM, suggesting that these drugs may reduce the pro-inflammatory state frequently observed in patients with T2DM [43]. GLP-1 RAs may reduce sepsis risk by suppressing inflammation, including cytokine production and monocyte adhesion, as shown in animal studies via STAT3 and NLRP3 pathways [44,45]. While direct clinical evidence is limited, our findings support these mechanisms, suggesting that GLP-1 RAs are a promising option for lowering sepsis risk in T2DM patients.

Similarly, the literature supports a protective effect of SGLT-2 inhibitors against sepsis in patients with T2DM, as demonstrated by our findings. SGLT2 inhibitors decrease glucose and sodium reabsorption in the proximal tubule, reducing intraglomerular pressure via enhanced tubuloglomerular feedback, thus alleviating glomerular hyperfiltration and improving renal hemodynamics in diabetic nephropathy [46,47,48]. SGLT-2i also mitigates oxidative stress and inflammation by modulating NADPH oxidases and protein kinase C, reducing reactive oxygen species and systemic inflammation, which benefits vascular stability and lowers infection risks in patients with T2DM [49,50,51].

Real-world data shows that SGLT-2i users have a lower incidence of pneumonia and sepsis, as well as reduced mortality associated with these conditions, compared with DPP-4i users [52]. Another study reported similar findings, with SGLT-2i associated with a 25% risk reduction in both pneumonia and severe sepsis as compared with DPP-4i [40]. However, by lowering the renal threshold for glucose reabsorption, SGLT-2i causes glycosuria, which creates a favorable environment for bacterial and fungal growth. This mechanism explains the increased incidence of urinary tract infections among SGLT-2i users [53]. In immunocompromised patients with T2DM and other comorbidities, even a localized urinary tract infection can progress to a systemic infection, increasing the risk of sepsis. This raises the concern that SGLT-2i may increase the risk of sepsis in patients with T2DM. However, contrary to the concerns about increased urinary tract infections during SGLT-2 I treatment in patients with T2DM [54], a multi-site cohort study observed a lower rate of urosepsis in SGLT2i users compared with DPP-4i users [55], which aligns with our findings.

In addition, the combination of SGLT2 inhibitors with insulin therapy has been suggested to yield favorable outcomes in terms of glycemic control while potentially minimizing the adverse effects traditionally associated with insulin, such as hypoglycemia, which can complicate the clinical picture in patients with T2DM [56,57]. By reducing insulin requirements and stabilizing glycemic fluctuations, SGLT2i-insulin combination therapy may help mitigate infection risk in patients with diabetes-related complications [58]. Similarly, combining GLP-1 RAs with basal insulin allows for better overall glycemic control, particularly in reducing postprandial glucose levels, a common area of concern in patients receiving insulin therapy [59]. Several studies indicate that this combination not only helps improve glycemic control but also reduces the associated risks typically linked to insulin alone, such as weight gain and hypoglycemia. For instance, adding a GLP-1 RA to basal insulin therapy can reduce the risk of hypoglycemia when compared with adding prandial insulin to the regimen [60]. This is particularly beneficial alongside insulin therapy for patients who have experienced difficulty managing their weight, as GLP-1 RAs can aid in weight loss due to their appetite-suppressing effects [61]. However, it is important to recognize that while the emerging data suggest potential benefits in this context, high-quality prospective studies directly comparing sepsis outcomes across treatment modalities in complicated T2DM populations remain limited.

This study is strengthened by its longitudinal design, use of real-world data, robust statistical analysis, and comprehensive covariate adjustment. The longitudinal approach enabled the assessment of temporal relationships between predictors and the outcome, enhancing the evidence for associations as compared with cross-sectional studies. Real-world data increased the generalizability of the findings by reflecting routine clinical practice across diverse patient populations. The time-adjusted multiple logistic regression model, adjusted for key confounders including age, gender, comorbidities, and laboratory parameters, ensured accurate estimates and minimized bias.

Although this study has notable limitations, its single-center approach may restrict the applicability of the findings beyond the current cohort. Being observational, it cannot establish causality, and unaccounted variables, such as dietary habits or socioeconomic status, could affect the outcomes. The use of ICD-10 codes and medical records may lead to inaccuracies in identifying comorbidities. The brief follow-up duration might not fully reflect the prolonged impact of antidiabetic drugs on sepsis risk. We also acknowledge the lack of data on medication adherence and dosage levels, which may influence the observed outcomes and should be considered in future research. These limitations suggest directions for future studies that should focus on prospective studies to confirm the association of SGLT2 inhibitors and GLP-1 Ras with reduced sepsis risk in T2DM patients. Exploring biomarkers such as procalcitonin or interleukins could improve risk stratification and guide personalized treatment strategies.

## 5. Conclusions

Our results demonstrate the significant influence of antidiabetic medications on sepsis risk in patients with T2DM. SGLT-2i and GLP-1 RAs showed protective effects after adjusting for key covariates, including age, gender, comorbidities, and laboratory parameters, suggesting their prioritization for high-risk patients. Conversely, insulin use is associated with increased risk, likely reflecting greater disease severity. These findings support more tailored antidiabetic treatments. SGLT-2 inhibitors and GLP-1 RAs may be preferred for older T2DM patients at elevated sepsis risk, while insulin-treated patients may benefit from closer infection monitoring. Incorporating sepsis risk into prescribing decisions could inform future guidelines and improve care pathways.

## Figures and Tables

**Table 1:** Baseline characteristics of patients with T2DM.

Variable		Baseline (% (*n*))
Age *		66 (60–74)
Gender	Female	50.9 (2550)
	Male	49.1 (2459)
BMI *		30.4 (26.1–35.7)
Comorbidities	Hypertension	62.7 (3141)
	Ischemic heart disease	39.1 (1956)
	Dyslipidemia	29.2 (1463)
	Atherosclerosis	27.7 (1388)
	Nephropathy	22.3 (1113)
	Neuropathy	12.4 (622)
Antidiabetic medications	Biguanides (metformin)	34.9 (1747)
	Insulin	23.5 (1179)
	SGLT-2 inhibitors	4.7 (219)
	GLP-1 RAs	3.9 (198)
Median blood glucose (mmol/L) *		7.8 (6.4–10.2)
Median HbA1C (%) *		7.2 (6.5–8.3)
Median C-reactive protein (mg/L) *		4.4 (2–11.1)
Median total cholesterol (mmol/L) *		4.6 (3.8–5.5)
Median triglycerides (mmol/L) *		1.6 (1.2–2.4)
Median creatinine (µmol/L) *		80 (65–101)

* median (IQR).

**Table 2:** Comparison of patients with sepsis and those without sepsis, noting baseline characteristics.

Variable	Category	Patients Without Sepsis	Patients With Sepsis	*p*-Value
Median age (IQR)		66 (60–73)	72 (64–79)	<0.001 **
Gender	Male	2362 (96.2%)	97 (3.8%)	0.916 *
	Female	2450 (96.1%)	100 3.9%)	
Median BMI (IQR)		30.4 (26.1–35.6)	34.5 (32.1–41.5)	0.006 **
Hypertension	Yes	2963 (94.3%)	178 (5.7%)	<0.001 *
	No	1849 (98.8%)	19 (1.2%)	
Ischemic heart disease	Yes	1861 (95.1%)	95 (4.9%)	0.007 *
	No	2951 (96.6%)	102 (3.4%)	
Dyslipidemia	Yes	1414 (96.6%)	49 (3.4%)	0.172 *
	No	3398 (95.8%)	148 (4.2%)	
Atherosclerosis	Yes	1314 (94.7)	74 (5.3%)	0.002 *
	No	3498 (96.6%)	123 (3.4%)	
Nephropathy	Yes	1001 (89.9)	112 (10.1%)	<0.001 *
	No	3811 (97.8%)	85 (2.2%)	
Neuropathy	Yes	603 (96.9%)	19 (3.1%)	0.229 *
	No	4209 (95.9%)	178 (4.1%)	
Metformin	Yes	1691 (96.8%)	56 (3.2%)	0.053 *
	No	3121 (95.7%)	141 (4.3%)	
Insulin	Yes	1093 (92.7%)	86 (7.3%)	<0.001 *
	No	3719 (97.1%)	111 (2.9%)	
SGLT-2 inhibitors	Yes	214 (97.7%)	5 (2.3%)	0.199 *
	No	4598 (95.9%)	192 (4.1%)	
GLP-1 RA	Yes	198 (100%)	0	**0.004**
	No	4614 (95.9%)	197 (4.1%)	
Median blood glucose (IQR), mmol/L		7.8 (6.3–10.1)	8.2 (6.4–10.7)	0.006 **
Median HbA1C (IQR), %		7.2 (6.5–8.3)	7.1 (6.1–8.5)	0.720 **
Median C-reactive protein (IQR), mg/L		4.1 (1.9–9.8)	60.1 (32.3–103.9)	<0.001 **
Median total cholesterol (IQR), mmol/L		4.6 (3.8–5.5)	4.1 (3.3–5.5)	<0.001 **
Median triglycerides (IQR), mmol/L		1.6 (1.2–2.4)	1.4 (1.0–1.9)	<0.001 **
Median creatinine (IQR), µmol/L		80 (65.5–100)	99 (67–161)	<0.001 **

* Chi square test (*n*, %), ** Wilcoxon rank–sum test, Bold values indicate statistical significance (*p* < 0.05), IQR: Interquartile rate.

**Table 3:** Time-adjusted multiple-logistic regression model.

Variable	Odds Ratio [95% CI]	*p*-Value
Gender (female/male)	1.13 [0.89–1.44]	0.289
Age (years)	1.02 [1.01–1.03]	**0.002**
Hypertension (no/yes)	3.4 [2.1–5.5]	**<0.001**
Ischemic heart disease (no/yes)	1.56 [1.22–2.0]	**0.001**
Nephropathy (no/yes)	1.98 [1.50–2.61]	**<0.001**
Metformin (no/yes)	1.07 [0.83–1.39]	0.587
Insulin (no/yes)	2.6 [2.09–3.34]	**<0.001**
SGLT-2 inhibitors (no/yes)	0.56 [0.34–0.91]	**0.02**
GLP-1 RAs (no/yes)	0.39 [0.19–0.79]	**0.009**
Median blood glucose (mmol/L)	1.07 [1.03–1.1]	**<0.001**
Median HbA1C (%)	0.88 [0.86–1.06]	0.136
Median C-reactive protein (mg/L)	1.04 [1.03–1.05]	**<0.001**
Median creatinine (µmol/L)	1.02 [1.01–1.03]	**<0.001**
Year: 2017/2016	1.13 [0.8–1.58]	0.471
Year: 2018/2016	0.97 [0.68–1.38]	0.876
Year: 2019/2016	1.06 [0.74–1.52]	0.724
Year: 2020/2016	1.32 [0.89–1.96]	0.153

Bold values indicate statistical significance (*p* < 0.05). Odds ratios are adjusted for variables in the model. AUC 0.819 [0.802–0.837].

## Data Availability

The datasets produced and/or analyzed in this study can be obtained from the corresponding author upon reasonable request.

## Abbreviations

The following abbreviations are used in this manuscript:

ACT	Anatomical therapeutic chemical
CI	Confidence interval
CVD	Cardiovascular diseases
DPP-4	Dipeptidyl peptidase-4 inhibitors
GLP-1 RA	Glucagon-like peptide-1 (GLP-1) receptor agonists
ICD-10	International Classification of Diseases-10
OR	Odds ratio
SGLT-2i	Sodium-glucose cotransporter-2 inhibitors
T2DM	Type 2 diabetes mellitus
